# Medication Literacy in a Cohort of Chinese Patients Discharged with Acute Coronary Syndrome

**DOI:** 10.3390/ijerph13070720

**Published:** 2016-07-15

**Authors:** Zhuqing Zhong, Feng Zheng, Yuna Guo, Aijing Luo

**Affiliations:** 1 Department of Social Medicine and Health Service Management, Xiangya School of Public Health, Central South University, Changsha 410013, China; zhongzhuqing2014@gmail.com; 2 Key Laboratory of Medical Information Research, Central South University, College of Hunan Province, Changsha 410013, China; 3 Department of Nursing, Third Xiangya Hospital, Central South University, Changsha 410013, China; 4 Department of Coronary Care Unit, Third Xiangya Hospital, Central South University, Changsha 410013, China; zhf89903811@163.com; 5 Shanghai International Peace Hospital, Shanghai 200030, China; gyuna@live.com

**Keywords:** acute coronary syndrome, discharged patients, medication literacy, medication

## Abstract

This study aims at investigating medication literacy of discharged patients with acute coronary syndrome (ACS) in China, and the important determinants of medication literacy among them. For this purpose, we conducted a prospective cohort study. Patient’s demographic and clinical data were retrieved from hospital charts and medication literacy was measured by instructed interview using the Chinese version of Medication Literacy Questionnaire on Discharged Patient between 7 and 30 days after the patient was discharged from the hospital. The results show that medication literacy for the surveyed patients was insufficient: >20% did not have adequate knowledge on the types of drugs and the frequency that they need to take the drugs, >30% did not know the name of and the dosage of the drugs they are taking, and >70% did not have adequate knowledge on the effects and side effects of the drugs they are taking. Our research indicated that medication literacy scores decreased with age but increased with education. The number of medicines the discharged patient took with them and days between discharge and interview were not associated with medication literacy levels.

## 1. Introduction

Medication literacy refers to the ability of the individual to obtain, appropriately interpret, and adequately handle the basic medication information, and make the right decisions accordingly [1]. It can serve as an important predictor of the correctness of medication behavior [2]. Previous studies found that many patients did not comply closely with the doctor’s orders after discharge from hospital, caused, at least in part, by the lack of medication literacy [3]. For example, a study in the United States found that medication error of non-hospitalized patient was at least 4 times that of hospitalized patients [4].

According to the 2014 report of cardiovascular disease in China, cardiovascular mortality ranked as the number one for cause of death among urban and rural residents, accounting for 44.8% deaths in the countryside and 41.9% deaths in the city [5]. Acute coronary syndrome is the main cause of Chinese adult heart disease hospitalization and mortality [6]. For patients with acute coronary syndrome (ACS), long-term, regular, and correct use of medication after discharge from hospital is critical to reduce the recurrence of major cardiovascular events [7,8]. Studies found in China found that the medication adherence of ACS patients was poor [9,10]. According to Hu et al., patients with ACS should fully follow the advice of the doctors and take antiplatelet drugs, β-blokers, angiotensin-converting enzyme inhibitors (ACEI)/angiotensin receptor antagonists (ARB), and statins, respectively, after discharge from hospital [11]. In contrast, treatment rates among discharged patients with ACS in China were only 10.6%, 10.1%, 7.6%, and 1.4%, respectively, for antiplatelet drugs, β-blokers, angiotensin-converting enzyme inhibitors (ACEI)/angiotensin receptor antagonists (ARB), and statins [11]. We have therefore undertaken this study to evaluate medication literacy in a cohort of discharged patients with ACS in China.

## 2. Experimental Section

### 2.1. Participants

Patients diagnosed with ACS at the Third Affiliated Xiangya Hospital of Central South University in Changsha, Hunan, China between March and June 2015 were invited to participate. Inclusion criteria were (1) age ≤85 years with competent language communication ability; (2) mentally stable; (3) voluntarily participating in the study under the principle of informed consent. Exclusion criteria were (1) engaged in health care related work currently or before retirement; (2) nonresponse (did not complete the questionnaire); (3) mentally unstable or major mental disorder; (4) major chronic diseases such as chronic obstructive emphysema, severe hepatic, or renal insufficiency; (5) there was a change in medication or health care provider between discharge and interview. All subjects gave their informed consent for inclusion before they participated in the study. We obtained ethical approval from third Xiangya Hospital, Central South University Research Ethics Board to conduct this study (project identification code: 2016-S001).

### 2.2. Data Collection

Medication literacy was measured by the Chinese version of Medication Literacy Questionnaire on Discharged Patient developed by Maniaci from Mayo Clinic in the United States [12]. Zheng et al. translated the questionnaire into Chinese and made modifications according to Chinese culture [13]. Specifically, in item 5, the original question was: “Were you prescribed any new medicines when you left the hospital?” Because in China no new drug was prescribed instead we asked patients bring drugs home. So we changed the question to: “Did you bring drugs with you when you discharged from hospital this time?” The questionnaire attempts to evaluate the patient’s ability to understand, calculate, and process pharmaceutical information. The questionnaire contains 9 items and uses a dichotomy scoring system, with a correct answer for a score of “1” and an incorrect answer for a score of “0”. At discharge the attending doctor provided instructions for the drugs to the patients, including the names, dosage, frequency of use, therapeutic effects, and main side effects. We compared the patient’s answers to the doctor’s instructions and if the answer was correct one score was given and if the answer is incorrect no score was given. Item 7 has only a “Yes” or “No” answer and item 9 has only specific names. Therefore, item 7 and item 9 contribute no score towards total score. As a result, the full score of this questionnaire is 7, with a higher score indicates higher level of medication literacy. For individual patients, the possible score is 0 to 7 without decimals, with 0 means all answers are incorrect and 7 means all answers correct. The mean was a summary of all patients in the subgroup. For example, a mean score of 6.67 means almost all answers are correct for all patients in this subgroup. The Cronbach’s α coefficient of the questionnaire content is 0.85, validity index is 0.81, and retesting reliability coefficient is 0.94 [13].

On the discharge date, patient’s demographic and clinical data such gender, age, education level, medication, and length of hospital stay were retrieved from hospital charts. Between 7 and 30 days after the patient was discharged from the hospital, staff at the research team made telephone calls to the patients to collect data on medication literacy.

### 2.3. Data Analyses

Means, standard deviations (SDs), and percentages were used to describe the patient’s baseline characteristics and medication literacy level. *T*-test was used in the univariate analysis and multiple linear regression analysis was used to analyze the independent effect of determinants of medication literacy. Full model with all six determinants considered in this study being entered into the multiple regression model. SPSS version 19.0 (2010, IBM SPSS Inc., New York, NY, USA) was used in all analyses.

## 3. Results

### 3.1. Medication Literacy of Participants

A total of 168 discharged patients with ACS were invited to participate in this study, 5 patients refused to participate in the study and 10 patients did not complete the questionnaire, leaving 153 (91%) for final analysis. Among the 153 patients, 104 were male and 49 were female. Means (SDs) of patient’s age were 63.2 (9.4) years, years of schooling were 8.3 (3.1) years, number of medicines that the patient were taking at discharge were 6.4 (1.4), hospital days were 9.5 (3.8), and days between discharge and interview were 11.2 (3.9).

Details of the medication literacy for this cohort of discharged patients with ACS are displayed in Table 1. In brief, mean (SD) of medication literacy score was 4.85 (1.52). Although all patients knew that they should take medicines after discharge from hospital, only 69.9% of them knew how many medicines they should take on daily basis, 59.5% could name the medicines they were taking, and about 20% knew the effects and side effects of the medications they were taking.

### 3.2. Determinants of Medication Literacy

Table 2 shows the results of univariate analysis. Four factors were significantly associated with medication literacy, with higher scores observed in male, younger, and highly educed patients, and patients who took a lower number of medicines at discharge.

Table 3 presents results from multiple regression analysis. Two factors showed independent association with medication literacy, with medication literacy scores decreased with age but increased with education.

## 4. Discussion

Our study, based on a cohort of discharged patients with ACS, found that medication literacy for these patients was insufficient: >20% did not have adequate knowledge on the types of drugs and the frequency that they need to take the drugs, >30% did not know the name of and the dosage of the drugs they are taking, and >70% did not have adequate knowledge on the effects and side effects of the drugs they are taking. As a result, it is critical to improve health literacy of these patients in China as improved literacy could improve medication adherence and therefore outcomes.

Two factors, namely age and education, were associated with medication literacy, with medication literacy levels decreased with age while increased with education. These findings were in general consistent with literacy [14,15]. Overall medication literacy level in this group of patients was much lower than previous reports [14,15]. The lack of medication literacy exposed these patients to increased risks of re-hospitalization, emergency department visits, or serious consequences due to adverse events related to unsafe medication [16,17].

The number of medicines the discharged patient took with them and the days between discharge and interview were not associated with medication literacy levels. This observation is consistent with the results of an earlier study [14,15]. On the other hand, age and education were associated with medication literacy, with increased medication literacy levels observed in younger and highly educated patients. These observations were also consistent with literature [18,19]. Although age may be related to opportunities of obtaining adequate education, we have adjusted each other of these two variables in the regression analyses to obtain an independent effect. It is possible that the cognitive abilities decline with age. It makes biological sense to assume these associations. When patients get old their cognitive ability tends to be lower and therefore they have lower ability to learn and remember. The strong and positive association between education level and medication literacy level suggests that literary ability in general can help better comprehend medication information, leading to high medication literacy. This observation further suggests that medication literacy could be improved by enforced patient education. Effective communication on medication between health care providers and discharged patients was the key to improving the medication literacy and ensuring the success of treatment after discharge [20].

Information communicated during hospitalization could provide guidance to patients and motivate them and allow them to notice particular points after discharge, so that they can take the initiatives to focus on medication information to reduce errors after discharge. In order to guarantee the effectiveness of communication, health care providers should first assess the medication literacy level of the discharged patients to predict their knowledge and behavior. Meanwhile, health care providers should also master the comprehensive knowledge of diseases, drug usages, and communication skills [21] to deal with patients with different levels of medication literacy.

In our hospital, patients did receive a discharge record, written education material, and guidance of medication from care providers before being discharged. Patients also often consulted with care providers on medication information during hospitalization. According to our record, 62.7% patients have been provided with information of the therapeutic and side effects of the medications before discharge. However, only 20.9% patients could correctly recall the therapeutic effects and only 21.6% patients could correctly recall the side effects of the medications they were taking. We speculate that patients could not fully capture and remember detailed medication information through casual verbal communication with health care providers. In addition, the written education material we provided did not satisfy the needs for some patients [22,23]. It is therefore important to provide high-quality and comprehendible written material for medication education, so that the patients can refer to the educational material after discharge on a regular basis.

There are several strengths in our study. First, although studies on medication literacy for patients discharged with ACS have been conducted in other populations, to the best of our knowledge, this is the first study that has measured the medication literacy and examined the determinants of medication literacy in ACS patients in a Chinese population. Because of the important differences in culture and health care systems in China as compared with other populations/jurisdictions, a study in the Chinese population is needed. Second, we used a validated tool to measure medication literacy, which lends validity to the study results. Third, patients surveyed in this study had homogeneous condition with solid diagnoses by a tertiary care center in China, which lends further validity to the study findings. Fourth, the collected data were analyzed by sophisticated statistical tools with output being easy to be interpret.

The limitations of our study should be acknowledged. Patients in this study were from a single hospital. Whether and to what extent they can represent other patient population in China remains to be determined. The sample size was also limited. Large scale studies in other jurisdictions in China are needed to see if our observations can be replicated in other Chinese populations.

## 5. Conclusions

We showed that medication literacy for discharged patients with ACS was insufficient. A deep communication between health care providers with patients at discharge and providing high-quality and comprehendible written material for medication education are important methods. However, further research is needed to find effective measures to decrease the risk to patients.

## Figures and Tables

**Table 1:** Medication literacy for discharged patients with acute coronary syndrome (ACS), Changsha, Hunan, China, March to June 2015 (*n* = 153).

Items	Number of Correct Answer	Proportion (%)
1. Did you take medicines after you were discharged from hospital?	153	100.0
2. How many kinds of medicine did you need to take every day?	107	69.9
3. Did you know the names of the medicines that you are taking?	91	59.5
4. Did you know the dosage of the medicines that you are taking?	86	56.2
5. Did you know how many times you should take the medicines?	105	68.6
6. Did you know the effects of every medicine that you are taking?	32	20.9
7. Have you ever been warned of the side effects of the medicines that you are taking?	96	62.7
8. Did you know the side effects of the medicines that you are taking?	33	21.6
9. Did you know whom you should consult with in case of questions related to the medicines you are taking?		
Local doctors	38	24.8
Doctors who give the prescription	59	38.6
Pharmacist	6	3.9
I don’t know	41	26.8
Others	9	5.9

**Table 2:** Results of univariate analysis of determinants of medication literacy for discharged patients with ACS, Changsha, Hunan, China, March to June 2015 (*n* = 153).

Variable Name	Mean (SD) of Total Score	*H/T*	*p*
Age		41.76	<0.01
≤40 years	6.67 ± 0.58		
41–50 years	6.43 ± 0.51		
51–60years	5.42 ± 1.02		
61–70years	4.51 ± 1.44		
>70 years	4.06 ± 1.70		
Gender		2.82	<0.01
Male	5.10 ± 1.40		
Female	4.33 ± 1.65		
Year of schooling		9.57	<0.01
≤9 years	3.91 ± 1.42		
>9 years	5.78 ± 0.95		
Interval between discharge and interview		1.94	0.06
≤7 days	4.27 ± 1.70		
>7 days	4.95 ± 1.48		
Number of medicines taken at discharge		3.02	<0.01
≤7	5.03 ± 1.45		
>7	4.13 ± 1.63		
Length of hospital stay		0.14	0.89
≤8 days	4.87 ± 1.45		
>8 days	4.83 ± 1.58		

*H*: Kruskal-wallis *H*; *T*: two-sample *t*-test.

**Table 3:** Results of multiple linear regression analysis of determinants of medication literacy for discharged patients with ACS, Changsha, Hunan, China, March to June 2015 (*n* = 153).

Determinants	*B*	*SE*	*p*
Age (each 10 years)	−0.04	0.01	<0.01
Education (each year of schooling)	0.22	0.04	<0.01
Hospital stay (each day of stay)	−0.06		0.17
Number of medicines	−0.11		0.09
Time between interview and discharge (each day from discharge)	0.11		0.09
Gender (male = 0 female = 1)	−0.05		0.42

*B*: Partial regression coefficient; *SE*: standard error.

## References

[1] Sauceda J.A., Loya A.M., Sias J.J., Taylor T., Wiebe J.S., Rivera J.O. (2012). Medication literacy in Spanish and English: Psychometric evaluation of a new assessment tool. J. Am. Pharm. Assoc..

[2] Kripalani S., Henderson L.E., Jacobson T.A., Vaccarino V. (2008). Medication use among inner-city patients after hospital discharge: Patient-reported barriers and solutions. Mayo Clin. Proc..

[3] Raynor D.K. (2009). Addressing medication literacy: A pharmacy practice priority. Int. J. Pharm. Pract..

[4] Tache S.V., Sonnichsen A., Ashcroft D.M. (2011). Prevalence of adverse drug events in ambulatory care: A systematic review. Annu. Pharmacother..

[5] Chen W., Gao R., Liu L., Zhu M., Wang W., Wang Y., Wu Z., Li H., Zheng Z., Jing L. (2015). The 2014 report of cardiovascular disease in China. Chin. Circ. J..

[6] Gao R. (2010). Guidelines for diagnosis and treatment of acute myocardial infarction. Chin. J. Cardiol..

[7] Rossi E., Perman G., Michelangelo H., Alonzo C.B., Brescacin L., Kopitowski K.S., Navarro Estrada J.L. (2014). Medication adherence to secondary prevention for coronary artery disease. Medicina (B Aires).

[8] Hu D., Sun Y. (2007). Optimization of drug therapy is the cornerstone of the secondary prevention of coronary heart disease. Chin. J. Intern. Med..

[9] Zhang M., Li Y. (2015). Comparison of standard secondary prevention and regular secondary prevention of ACS (acute coronary syndrome) patients: 12-Month follow-up result. China Med. Pharm..

[10] Li S., Li D., Lu Y., Chen S., Qin Y., Zhao X. (2014). Secondary prevention following percutaneous coronary intervention in patients with acute coronary syndrome: A survey and analysis. Acad. J. Second Mil. Med. Univ..

[11] Chen Y., Li L., Zhang Q., Clarke R., Chen J., Guo Y., Bian Z., Pan X., Peto R., Tao R. (2014). Use of drug treatment for secondary prevention of cardiovascular disease in urban and rural communities of China: China Kadoorie Biobank Study of 0.5 million people. Int. J. Cardiol..

[12] Maniaci M.J., Heckman M.G., Dawson N.L. (2008). Functional health literacy and understanding of medications at discharge. Mayo Clin. Proc..

[13] Zheng F., Ding S., Zhong Z., Pan C., Xie J., Qin C. (2015). Investigation on status of discharged patients’ medication literacy after coronary artery stent implantation. Chin. Nurs. Res..

[14] King J.L., Schommer J.C., Wirsching R.G. (1998). Patients’ knowledge of medication care plans after hospital discharge. Am. J. Health Syst. Pharm..

[15] Marks J.R., Schectman J.M., Groninger H., Margaret L. (2010). The association of health literacy and socio-demographic factors with medication knowledge. Patient Educ. Couns..

[16] Gandhi T.K., Weingart S.N., Borus J., Seger A.C., Peterson J., Burdick E., Seger D.L., Shu K., Federico F., Leape L.L. (2003). Adverse drug events in ambulatory care. N. Engl. J. Med..

[17] Toren O., Kerzman H., Koren N., Baron-Epel O. (2006). Patients’ knowledge regarding medication therapy and the association with health services utilization. Eur. J. Cardiovasc. Nurs..

[18] Davis T.C., Wolf M.S., Bass P.F., Thompson J.A., Tilson H.H., Neuberger M., Parker R.M. (2006). Literacy and misunderstanding prescription drug labels. Ann. Intern. Med..

[19] Davis T.C., Wolf M.S., Bass P.F., Middlebrooks M., Kennen E., Baker D.W., Bennett C.L., Durazo-Arvizu R., Bocchini A., Savory S. (2006). Low literacy impairs comprehension of prescription drug warning labels. J. Gen. Intern. Med..

[20] Raynor D.K. (2008). Medication literacy is a 2-way street. Mayo Clin. Proc..

[21] Wouda J.C., van de Wiel H.B. (2012). The communication competency of medical students, residents and consultants. Patient Educ. Couns..

[22] Raynor D.K., Blenkinsopp A., Knapp P., Grime J., Nicolson J., Pollock K., Dorer G., Gilbody S., Dickinson D., Maule A.J. (2007). A systematic review of quantitative and qualitative research on the role and effectiveness of written information available to patients about individual medicines. Health Technol. Assess..

[23] Duggan C., Bates I. (2008). Medicine information needs of patients: The relationships between information needs, diagnosis and disease. Qual. Saf. Health Care.

